# Resistome flow in global landfill systems

**DOI:** 10.1002/imo2.70075

**Published:** 2026-01-06

**Authors:** Wenqing Hong, Rui Zhang, Xiaoxing Lin, Yangqing Wang, Shu Yang, Farees ud din Mufti, Yuping Yao, Guowei Zhou, Chuanwu Xi, Weixi Shu, Gordon Price, Youcai Zhao, Liyan Song

**Affiliations:** ^1^ School of Resources and Environmental Engineering Anhui University Hefei China; ^2^ School of Engineering Westlake University Hangzhou China; ^3^ Chongqing Institute of Green and Intelligent Technology Chinese Academy of Science Chongqing China; ^4^ Institute of Public Health Sciences, Division of Life Science and Medicine University of Science and Technology of China Hefei China; ^5^ Department of Environmental Health Sciences University of Michigan Ann Arbor Michigan USA; ^6^ Department of Engineering Dalhousie University Halifax Nova Scotia Canada; ^7^ The State Key Laboratory of Pollution Control and Resource Reuse, School of Environmental Science and Engineering Tongji University Shanghai China

**Keywords:** landfill systems, multidrug resistant genes, One Health, pathogens, resistome, resistome flow

## Abstract

Antibiotic resistance genes (ARGs) represent a critical global public health threat. Effective resistome management within the One Health framework requires a comprehensive understanding of the sources and movement of ARGs across environmental media. This study provides a detailed analysis of resistome characteristics and dynamics in global landfill systems. Landfill systems were found to harbor diverse and highly mobile ARGs, with multidrug‐resistant genes (MDRGs) comprising 32.63% of total ARGs, and comparable or even higher levels of associated ARG subtypes than in other environmental media, such as acidic mine wastewater. The ARG density on plasmids was 3.5 times higher than that on chromosomal sequences, with five times more abundant ARG‐carrying plasmid‐associated contigs in landfill systems than that in wastewater. At the metagenome‐assembled genome level, members of the phylum Pseudomonadota were identified as the most abundant hosts (29.72%), harboring 47.74% and 56.47% of the total ARGs and MDRGs, respectively. Genomic analysis of 35 *Pseudomonas* spp. strains revealed unique *Pseudomonas* species in landfill systems, including *P. aeruginosa*. Resistome flow pathways were further mapped, including inflows into landfill (via soil, wastewater, freshwater, and the human and pig gut), movement within landfills (across landfill refuse, leachate, and airborne particles), and outflows from landfills (via landfill leachate effluent, gas emissions, and refuse evacuation). The results emphasized diverse resistome development and movement within landfill systems, ultimately contributing to the environmental spread of ARGs. Thus, our study highlights the urgent need for comprehensive policies and management strategies to mitigate resistome proliferation at every stage of municipal solid waste management.

## Highlights

- Landfill systems serve as hotspots of antibiotic resistance genes (ARGs), including dominant multidrug‐resistant genes.
- The density of ARGs on plasmid was 3.5 times higher than that on chromosomal.
- Resistant flows in landfill systems were mapped in conjunction with one health.
- Global landfill systems serve as flow nodes for resistome.

## Graphical abstract

Global landfill systems represent key hubs of resistome, serving as dynamic reservoirs and amplifiers through plasmid‐driven gene mobility. Waste flows and environmental pathways sustain the transboundary resistome networks, accelerating resistance dissemination across ecosystems. Addressing this challenge requires reclassifying landfill systems as integral to One Health frameworks, driving policy innovations to mitigate resistome proliferation across waste lifecycles, from household disposal to post‐treatment care.

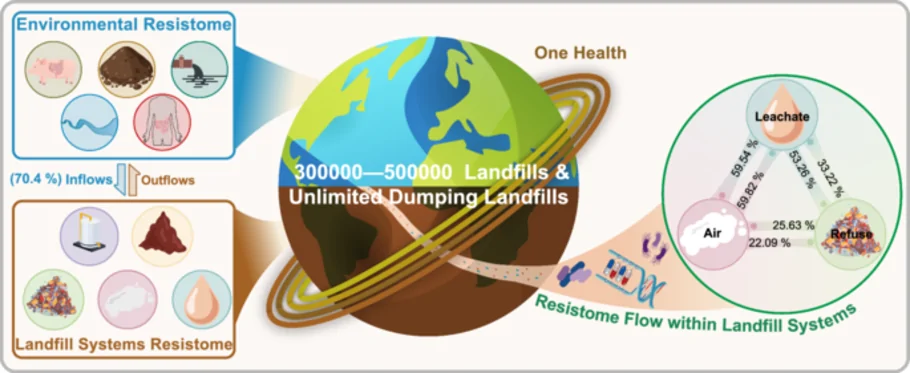

## 1 INTRODUCTION

The One Health Joint Plan of Action (2022−2026), developed collaboratively by the World Health Organization (WHO), Food and Agriculture Organization, United Nations Environmental Program (UNEP), and World Organization of Animal Health, seeks to address current and future health challenges through a multisectoral and multilevel framework that integrates humans, animals, and environmental health [1, 2]. Recognizing the interconnections among humans, animals, plants, and ecosystems across spatial and temporal scales, is crucial for preventing, identifying, and managing potential health risks [3]. By 2050, antibiotic‐resistant bacteria are projected to cause up to 10 million deaths and economic losses of $100 trillion annually [4, 5]. Identifying the spread of antibiotic resistance genes (ARGs) within this socio‐ecological system is a public health priority [6, 7]. The One Health framework emphasizes the importance of improving prevention and response strategies for ARG‐associated health threats, thereby enhancing the health and sustainability of environments characterized by complex organismal interactions and pollution [8, 9].

Public health and safety are global concerns, particularly in environments contaminated with ARGs, such as soil [10], mines [11], wastewater treatment plants [12], and landfills [13]. Although ARGs are increasingly reported in municipal solid waste (MSW) landfills, standardized and publicly available metagenomic data remain scarce, and landfill microbiomes, resistomes, and viromes remain largely unexplored at the global scale, especially when compared to sources such as soil [10], wastewater [12], and freshwater [14]. This oversight can be attributed to two primary factors. First, landfills are often viewed as being peripheral to city centers, reinforcing the misconception that their impact on human health is negligible. Indeed, by serving as a city's gut by processing its solid waste, the metabolic activity of landfills can substantially impact the health of its residents. Second, the high cost associated with deep‐layer refuse sampling poses considerable logistical and financial barriers that prevent comprehensive studies from being conducted [15]. However, the importance of addressing landfill resistomes cannot be overstated. Global MSW production is projected to increase from 2.1 to 3.8 billion tons from 2023 to 2050 [16], with over half of this waste deposited in an estimated 300,000−500,000 landfills systems worldwide [17, 18]. Landfills are not merely waste repositories but also act as hubs for accumulating anthropogenic contaminants, such as unused and unwanted pharmaceuticals and per‐ and polyfluoroalkyl substances [19]. Consequently, landfills can act as substantial sources of ARGs, contributing to the resistomes of surrounding environments containing high concentrations of antibiotics and other stressors, such as heavy metals [5]. Additionally, ARGs in landfills can enter the surrounding environment via aerosols, groundwater contamination, worker exposure, wildlife spread, and soil pollution [5], facilitating resistome integration and sustaining a destructive cycle of contamination and resistance proliferation. Addressing this critical issue is essential to mitigating the ongoing threats posed by landfill resistomes.

This study employs metagenomic data from global landfill systems to study the distribution and potential risks associated with ARGs and their pathogenic hosts, which were identified based on the principle of human pathogenic bacteria (HPB) identification. Additionally, resistome flow pathways were mapped by tracing the movement of ARGs into, within, and out of landfill systems to better understand their roles in resistome dissemination. Herein, a landfill system is defined as a primary landfill component during the MSW lifecycle, including landfill refuse, airborne particles, and leachate, MSW decomposition and leachate recirculation bioreactors, and activated sludge from leachate treatment facilities. Results indicated that landfill systems exhibit unique resistome characteristics, including a high ARG load (40,442 total predicted ARG‐like open reading frames (ORFs)), a predominance of multidrug‐resistant genes (MDRGs) (36.37% ± 6.51% of total ARGs), high mobility, and a predominance of ARG‐hosting bacteria from the phylum Pseudomonadota (29.72%), notably, *Pseudomonas aeruginosa*. The study highlights severely underestimated risk associated with ARGs in global landfill systems, emphasizing the urgent need for targeted policies and interventions to address this critical public health concern.

## 2 RESULTS

### 2.1 MDRGs are the main ARGs

The five landfill components spanning the MSW lifecycle within a landfill are illustrated in Figure 1A. The landfill system dataset comprised 161 metagenomic samples on a global scale, including 43 samples from our laboratory and 118 samples from public databases (Table S1). This dataset covered 21 cities across 7 countries, comprising the most comprehensive dataset currently available for assessing the potential risk posed by landfill systems within the human urban landscape (Figure S1A).

**Figure 1:**
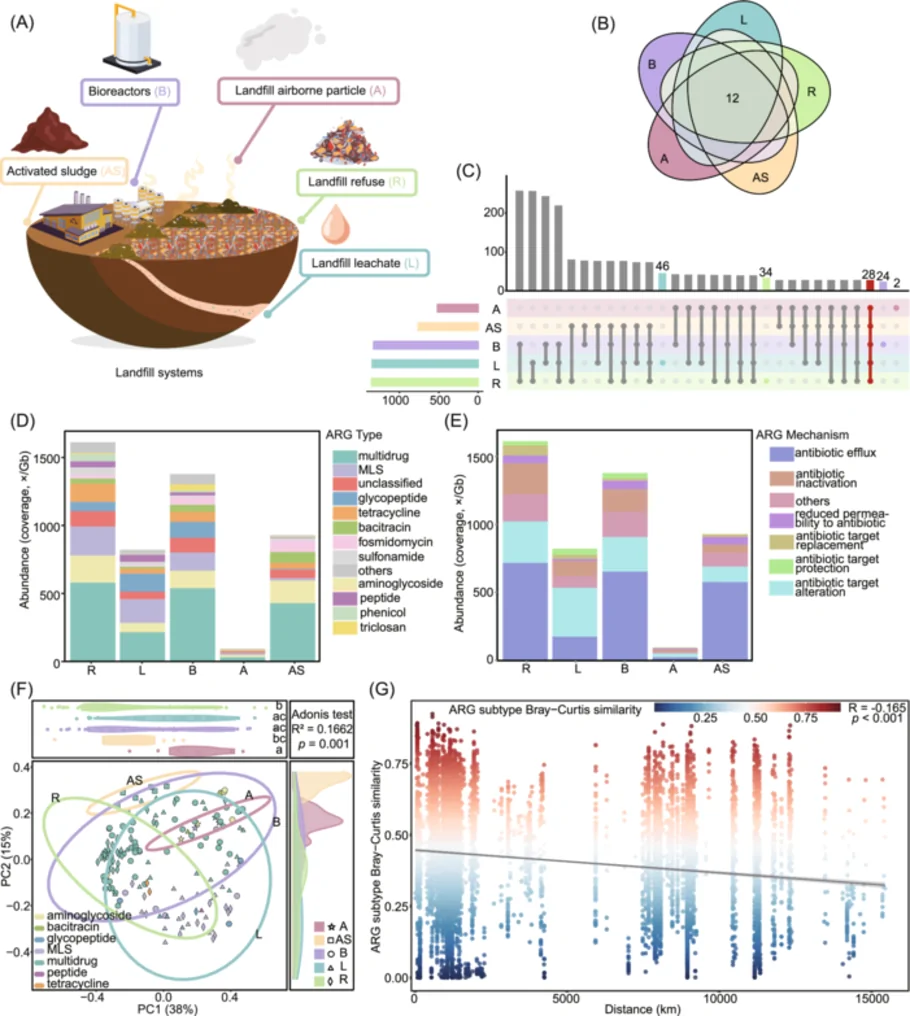
ARG distribution in global landfill systems. (A) Landfill systems, including landfill refuse, airborne particles, and leachate, MSW decomposition and leachate recirculation bioreactors, and activated sludge from leachate treatment facilities. Distribution and sharing of ARG types (B) and subtypes (C) among five habitats in landfill systems. Abundance (×/Gb) and distribution pattern of the top 12 ARG types (D) and ARG mechanisms (E) among five habitats in landfill systems. (F) PCoA analysis of ARG distribution, the Adonis test revealed that there were differences in the distribution patterns of ARG types among the five habitats of the landfill system. In the figure, the color of each sampling point represents the ARG type with the highest abundance (×/Gb) at that point. (G) Bray–Curtis similarity analysis of ARG subtype distribution with geographic distance. R, landfill refuse; A, landfill airborne particles; B, MSW decomposition and leachate recirculation bioreactors; L, landfill leachate; ARG, antibiotic resistance gene; AS, activated sludge from leachate treatment facilities; MLS, macrolide‐lincosamide‐streptogramin; PCoA, principal coordinate analysis.

Across global landfill systems, 40,442 ORFs were annotated as ARGs, comprising 22 ARG types and 428 ARG subtypes. Among them,12 ARG types and 28 ARG subtypes were shared across the five landfill components (Figure 1B,C). ARG abundance, measured in units of coverage (×/Gb), varied across landfill components, with landfill refuse harboring the highest abundance (1612.5×/Gb) and landfill airborne particles harboring the lowest abundance (93.9×/Gb) (Figure 1D,E). The Adonis test indicated that the composition of ARG types and subtypes significantly differed across landfill components (*p* < 0.05; Figure 1F and Figure S2), and significant heterogeneity of group dispersions was also detected (Betadisper, *p* < 0.05).

The ARG types with the highest relative abundance were MDRGs (36.37% ± 6.51%), aminoglycoside resistance genes (12.87% ± 3.82%), and macrolide‐lincosamide‐streptogramin resistance genes (12.89% ± 6.76%). MDRGs were overwhelmingly dominant in 68.32% of samples, accounting for 32.63% ± 13.60% of total ARGs (Figure 1D). Among ARG subtypes, the most abundant were *rpoB2* (7.71% ± 4.83%), *ompR* (4.88% ± 2.61%), and *ArlR* (4.50% ± 1.94%) (Figure S3). Antibiotic efflux and antibiotic target alteration were responsible for 39.88% ± 15.23% and 24.79% ± 11.18% of antibiotic resistance mechanisms in landfill systems, respectively. Notably, 73% of ARGs within the MDRG category were associated with multidrug efflux pumps. The distribution of ARG subtypes exhibited a significant decay trend with increasing geographical distance (*p* < 0.001; Figure 1G), indicating that the spread of ARGs is geographically constrained. Additionally, the Chao and Shannon indices of ARGs demonstrated a significantly decreasing trend with higher latitude (*p* < 0.001; Figure S1B). Random forest analysis identified *mexA* as a specific ARG within landfill systems. The abundance of *mexA* was significantly and positively correlated with the total abundance of ARGs in landfill systems (*R*² = 0.552, *p* < 0.001; Figures S4 and S5), supporting this finding. While these results were statistically significant, studies with additional landfill samples and explanatory variables are warranted to further support these findings.

### 2.2 Higher ARG density on plasmid‐associated contigs

The location of a gene is known to determine its mobility, with plasmids being more mobile than chromosomes [20]. A total of 38,047 ARG‐carrying contigs were detected within landfill systems, among which 9419 (24.8%) were predicted to be plasmid sequences and 16,479 (43.3%) were classified as chromosomal sequences (Figure 2A). Regarding ARG abundance (coverage, ×/Gb), 19.3% and 57.3% of ARGs were associated with plasmids and chromosomes, respectively (Figure 2B). The ARG density was calculated as the number of ARGs per 100 kb, following established methodologies in environmental resistome research [11]. Although chromosomes carried a greater number and higher abundance of ARGs, plasmids had a higher ARG density, averaging 31 ARGs per 100 kb with an abundance of 126.4 per 100 kb, in contrast to chromosomes, which carried an average of 5 ARGs per 100 kb and an abundance of 35.7 per 100 kb. In addition, plasmid‐mediated ARGs exhibited high overall abundances of sulfonamide, phenicol, diaminopyrimidine, beta‐lactam, and nucleoside resistance genes, comprising 58.9%, 56.6%, 40.6%, 38.3%, and 32.8% of the total ARG abundance, respectively (Figure 2A). Also, sulfonamide resistance genes comprised >50% of all plasmid‐mediated ARGs.

**Figure 2:**
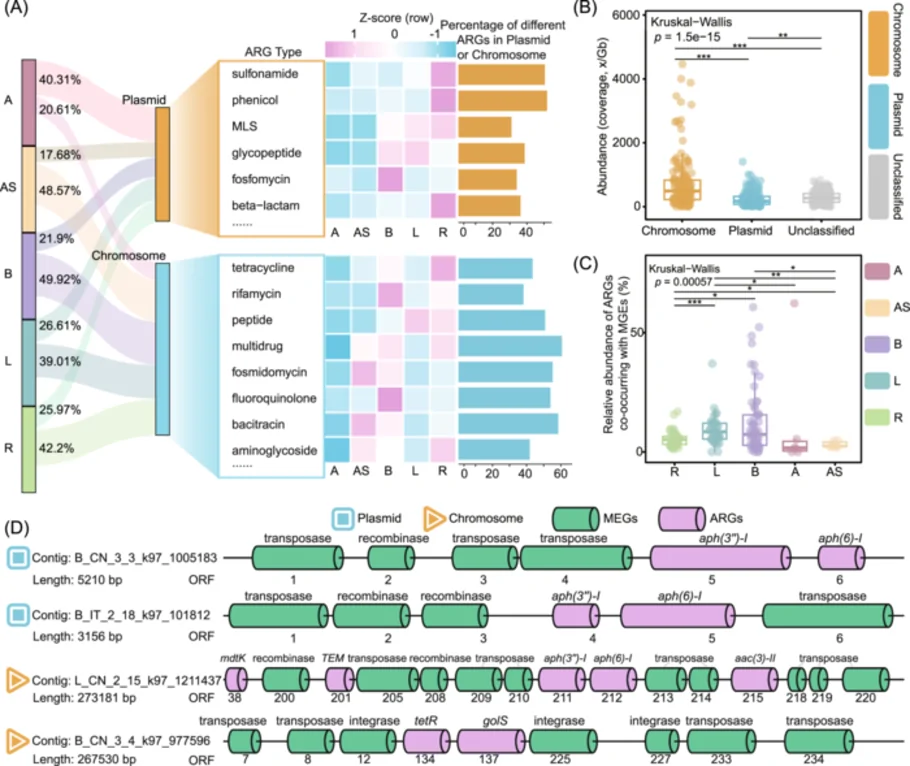
Mobility of ARGs in global landfill systems. (A) ARGs located on plasmids or chromosomes and their abundances across five landfill components. (B) Variation in the abundance of ARGs located on chromosomes or plasmids. (C) Variation in the relative abundance of ARGs co‐occurring with MGEs across five landfill components. (D) Schematic diagram of contigs containing ARGs and MGEs. *p < 0.05, **p < 0.01, ***p < 0.001. R, landfill refuse; A, landfill airborne particles; B, MSW decomposition and leachate recirculation bioreactors; L, landfill leachate; AS, activated sludge from leachate treatment facilities. ARG, antibiotic resistance gene; MGE, mobile genetic element; ORF, open reading frames.

Another ARG mobility‐enhancing mechanism is their co‐occurrence with mobile genetic elements (MGEs), where ARGs found within the same sequence as MGEs are considered to be more mobile than their counterparts that are not associated with MGEs [21]. Procrustes analysis demonstrated that ARG distribution was significantly correlated with MGE composition (*M* ^2^ = 0.929, *p* < 0.001; Figure S6), suggesting the potential presence of highly mobile ARG types in landfill systems. Co‐occurring ARGs and MGEs comprised 12.2% of the total ARG abundance, with triclosan‐associated ARGs exhibiting the highest co‐occurring rate (67.8%), followed by mupirocin resistance genes (50.1%) and sulfonamide resistance genes (32.6%) (Figure 2C). Meanwhile, the abundance of ARGs co‐occurring with MGEs was 24,604.26×/Gb, some of which were located on contigs predicted to be plasmids, suggesting their heightened mobility (Figure 2D). Despite chromosomes hosting 2–3 times more ARGs and MEGs than plasmids, their sequence lengths were 50–100 times longer than those on plasmids (Figure 2D).

### 2.3 Evidence of ARG transmission from inputs to effluents

MDRGs pose a substantial threat to human health owing to their ability to transfer between pathogenic bacteria in clinical and environmental settings, leading to the emergence of multidrug‐resistant infections. To investigate potential environmental sources of MDRGs in landfill systems, the Sourcetracker2 tool was applied to the global metagenome dataset from Global Microbial Gene Catalog (https://gmgc.embl.de/), focusing on sources with clear pathways to landfills. Sourcetracker2 employs a Bayesian algorithm to predict ARG sources based on the similarity of community structures between source and sink samples [22]. Human gut microbes are disseminated via sewage, with the resulting sludge deposited in landfills. Correspondingly, pig farming (with production projected at 130 million metric tons by 2034) generates vast volumes of manure that contaminate soil and water, which may eventually reach landfill leachate [23]. Herein, each landfill component was considered to be an independent system. Complex sources of secondary MDRGs were not explored. Results indicated that the MDRGs in landfill systems were attributed to soil (34.6%), the pig gut (24.2%), wastewater (4.7%), freshwater (4.5%), and the human gut (2.4%), with the remaining 29.6% originating from unknown sources (Figure 3A). Additionally, construction of a network of shared MDRGs linking “source‐sink” systems revealed that the average connection strength corroborated the Sourcetracker2 findings: MDRGs attributed to soil and the pig gut exhibited the highest percentages of the total ARG abundance (Figure S7).

**Figure 3:**
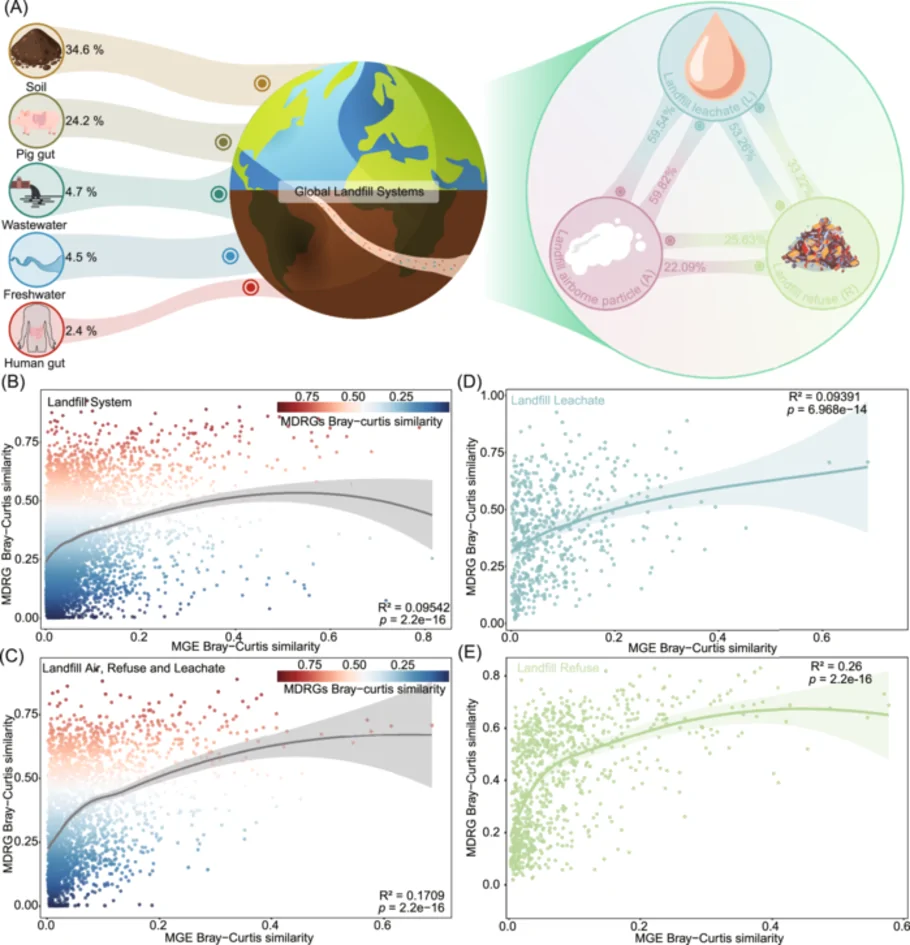
MDRG flow in global landfill systems. (A) MDRG flows into landfill systems as predicted by SourceTracker2 (left) and within landfill systems (right), with dots indicating receivers, and numbers and colors representing senders. (B, C) Correlation analysis between Bray–Curtis similarities of MGEs and MDRGs in landfill systems and the triphasic environment within landfill systems (landfill airborne particles, refuse, and leachate. (D, E) Correlation analysis between Bray–Curtis similarities of MGEs and MDRGs in landfill leachate and refuse. MDRG, multidrug‐resistant gene; MGE, mobile genetic element.

Landfill systems serve as “sinks” for MDRGs, emphasizing the need to investigate their movement within these systems [13, 19, 24, 25]. Herein, stronger correlations were observed between MDRGs and MGEs in three landfill phases (*R* ^2^ = 0.171, *p* < 0.001) compared to overall landfill systems (*R* ^2^ = 0.096, *p* < 0.001) (Figure 3B–E). SourceTracker2 was employed to identify MDRG flow across the solid, liquid, and gas phases of landfill systems (Figure 3A). MDRGs in landfill leachate exhibited the highest level of interaction with other environments, with 59.54% and 53.26% of MDRGs in landfill airborne particles and refuse originating from landfill leachate, respectively. Specifically, the correlation between MDRGs and MGEs was stronger in refuse (*R* ^2^ = 0.094, *p* < 0.001) than in leachate (*R* ^2^ = 0.26, *p* < 0.001) (Figure 3B–E). During the resistome flow process, the distribution of a specific ARG (*mexA*) across the solid‐liquid‐gas triphasic environment within landfill systems generally aligned with the trend of total ARG abundance in landfill systems (Figure S8).

### 2.4 Bacterial phylum pseudomonadota dominates resistome carriers

Identification of ARG hosts within landfill systems is critical for understanding ARG propagation and managing associated health risks. Herein, ARG hosts were identified through metagenome‐assembled genomes (MAGs) in landfill systems. A total of 1537 non‐redundant, high‐quality MAGs (≥90% completeness and <5% contamination) were recovered from the global landfill metagenomics collection (Table S2). Among these, 868 MAGs (56.5%) were found to carry ARGs, which were classified into 46 phyla, with the phylum Pseudomonadota having the highest count (258) and total abundance (2018.4×/Gb) of MAGs (Figure 4A,B). In total, MAGs carried 3025 ARGs, representing 20 ARG types and 243 subtypes, with 527 MAGs (60.7%) carrying multiple ARGs. Notably, the phylum Pseudomonadota is a major carrier of ARGs not only in the environment but also within the intestinal microbiome, as reported by Feng et al. [26].

**Figure 4:**
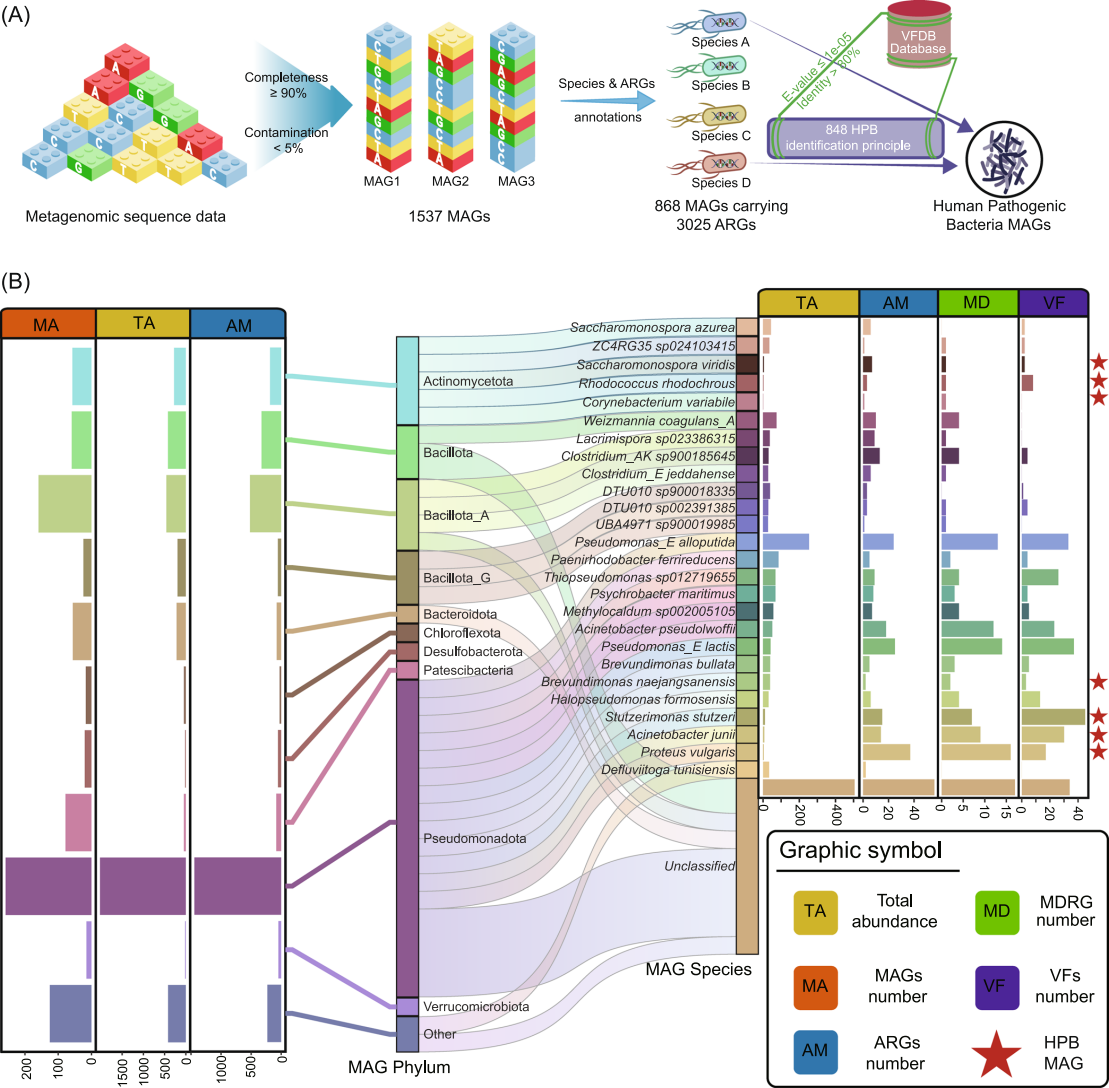
MAGs in global landfill systems. (A) MAG assembly and HPB identification process: 1537 MAGs were recovered from metagenome sequences of global landfill systems. Following species annotation, MAGs were identified as HPBs through the HPB identification principle and further confirmed through VFDB annotation of VFs. (B) (Left) Top 10 phyla, ranked by total abundance, are depicted from left to right, and remaining taxa are categorized as “other”; (Right) Corresponding species‐level phyla. The outer bar represents the total abundance, total MAG count, ARG‐carrying MAGs, VFDB‐annotated VFs, and potential HPBs identified through the HPB identification principle for phylum and species taxa. MAG, metagenome‐assembled genome; VFDB, virulence factor database; HPB, human pathogenic bacteria; ARG, antibiotic resistance gene; MDRG, multidrug‐resistant gene; VF, virulence factors.

Among the 3025 ARGs, 1376 (45.5%, total abundance of 2065.7×/Gb) were classified as MDRGs, comprising the most prevalent ARG type in landfill systems. Members of the phylum Pseudomonadota harbored 47.74% and 58.13% of the total ARGs and MDRGs, respectively, with 777 MDRGs identified within this group. Specifically, MAG_1073, belonging to the species *Proteus vulgaris* within the phylum Pseudomonadota, harbored the highest number of ARGs, with 37 ARGs covering 11 different ARG types (Figure 4B).

MDRGs are often strongly associated with HPB, such as *Pseudomonas aeruginosa* [27]. The HPB identification principle, based on 848 common HPBs, was adopted to identify HPB genomes among the identified MAGs. The MAG species annotations were then compared with the HPB list, and the presence of virulence factors (VFs) was determined based on virulence factor database (VFDB) annotations (Figure 4A). MAGs annotated as HPB species and carrying VFs were classified as potentially pathogenic bacterial MAGs. Among the revised data, four MAGs were affiliated with the phylum Pseudomonadota and two MAGs were associated with the phylum Actinomycetota (Figure 4B). Pseudomonadota‐associated MAGs carried a total of 34 MDRGs and 95 VFs (Figure 4B). VF abundance represented potential pathogenicity, with *P. vulgaris* exhibiting high abundances of ARGs and MDRGs, although it may not be as pathogenic as *Stutzerimonas stutzeri* [28]. The combined abundance of these pathogens (*S. stutzeri*, *junii*, *Saccharomonospora viridis*, *P. vulgaris*, *Brevundimonas naejangsanensis*, and *Rhodococcus rhodochrous*) in landfill refuse and bioreactor samples accounted for over 90% of their total abundance across all five habitats (Figure S9). Phylum Pseudomonadota has been identified as the primary host of ARGs in global landfill system datasets. Consequently, *Pseudomonas* spp. was selected as a model taxon to investigate the microscale transmission mechanisms of its associated ARGs. Then, we combined an in‑depth analysis of strains isolated from Chinese landfills with publicly available whole‑genome data from global sources.

### 2.5 *Pseudomonas* spp. genomes

The phylum Pseudomonadota (258 MAGs, 29.72%) predominated the microbiota in global landfill systems, aligning with our previous findings that *Pseudomonas* spp. are abundant in landfills [13, 19, 24, 25]. *Pseudomonas* spp. is known as conditionally pathogenic bacterium, commonly responsible for clinical infections and displaying significant multidrug‐resistant [27]. Herein, members of the phylum Pseudomonadota harbored the highest number of MGEs (38,293) (Figure S10). Thirty‐five *Pseudomonas* spp. strains were screened to obtain high‐quality, genome‐wide data, including 3 strains isolated from our lab, 19 from global landfill systems, and 13 strains isolated from the human gut (Table S3).

The isolated *Pseudomonas* spp. strains harbored 83 ARGs, including *adeF*. Among them, 53 ARGs were found in *Pseudomonas* spp. isolated from landfill systems, while 16 ARGs were found in *Pseudomonas* spp. isolated from the human gut. Notably, 9 ARGs were unique to *Pseudomonas* spp. in landfill systems, including *blaZ* and *OXA‐494*, which encode β‐lactamases, these genes are associated with clinically important antibiotics such as penicillin and cephalosporins. While 8 ARGs were exclusive to *Pseudomonas* spp. in the human gut, where *vanX* is one of the key components of vancomycin resistance (Table S4). These unique ARGs suggest an underestimated pathway for ARG circulation between environmental and clinical sources. A significant difference in MGE content was observed between *Pseudomonas* spp. in the human gut and landfill systems (*p* = 0.018). Despite the larger genome size of *Pseudomonas* spp. in landfill systems, the genome of *Pseudomonas* spp. in the human gut contained a greater number of ARGs (Figure S11), suggesting a greater frequency of gene transfer in the human gut than in landfill systems (Figure 5 and Figure S11). Notably, *P. saudiphocaensis* strains, P1_w and P2_w, isolated from Chinese landfill refuse and leachate samples, exhibited different distribution patterns across their genome characters, including genomic islands, ARGs, and MEGs. Phylogenetic analysis revealed that the first category on the phylogenetic tree, ranging from GMBC10023417 to GMBC10016245, was identified as *Pseudomonas aeruginosa*. This species is classified as a high‐priority pathogen in the WHO's Bacterial Priority Pathogens List [4]. Compared to the genomes of other *Pseudomonas* spp. strains, the *P. aeruginosa* genome was characterized by higher abundances of MGEs, VFs, MDRGs, and ARGs, with a similar size. Moreover, the *P. aeruginosa* genome contained more ARGs associated with the efflux pump mechanism (Figure 5), with the clusters of orthologous groups annotation assigning genes predominantly within the metabolism category (1882.75 ± 18.67) (Figure S12). In addition, the ratio of MDRGs to ARGs for *P. aeruginosa* was statistically higher in the human gut than in the landfill systems, although the numbers of ARGs and genomic islands were lower in the human gut than in landfill systems (Figure S13). *adeF*, identified in *P. aeruginosa*, is a typical high‐risk efflux pump gene. The abundance of genomic islands, defined as a cluster of genes that can be horizontally transferred within microbial genomes, did not significantly differ between *Pseudomonas* spp. in the human gut and landfill systems (Figure 5 and Figure S12).

**Figure 5:**
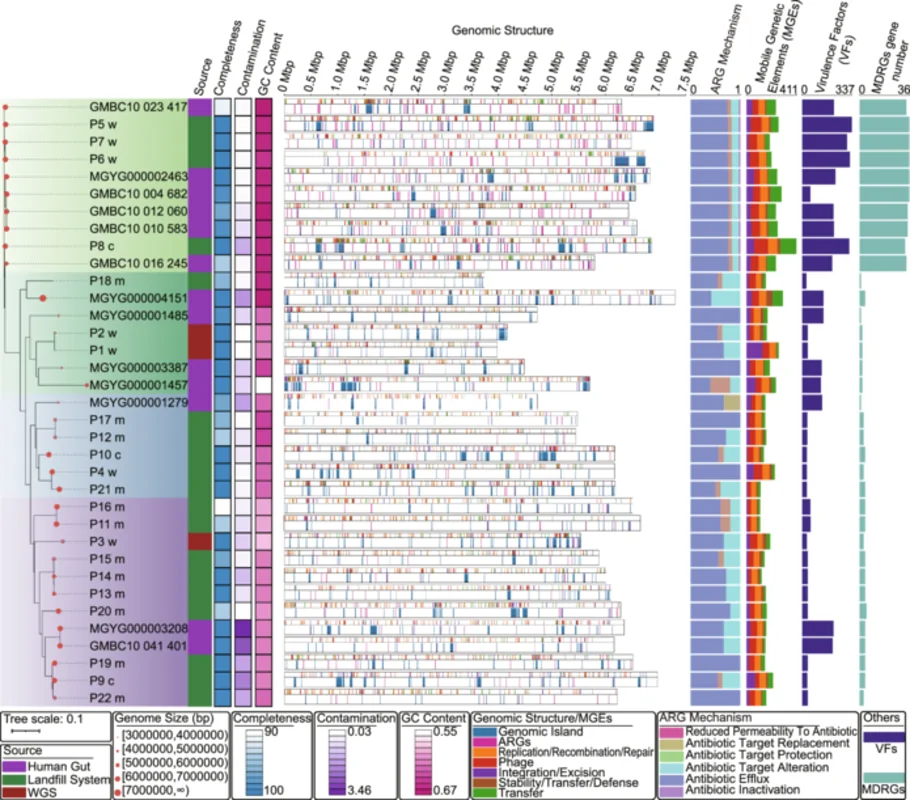
Genomic information on 35 *Pseudomonas* spp. strains. (Left) Genomes classified by color based on phylogenetic relationships. Points on the phylogenetic tree represent genome size (bp), while “source” indicates the source of genome collection for each strain. Genome completeness, contamination levels, GC content, and annotation details are provided below the figure. (Right) Bar charts illustrating the proportions of resistance mechanisms from ARGs, numbers of MGEs, and counts of VFs and MDRGs within strain genomes. Bp, base pairs; ARG, antibiotic resistance gene; MDRG, multidrug‐resistant gene; VF, virulence factors; MGE, mobile genetic element; WGS, whole genome sequencing.

### 2.6 ARG profile comparison in representative environmental media

ARG distribution varies across environmental media, which is determined by the specific characteristics of each environment [29, 30, 31]. We compared the prevalence of ARGs in our landfill system with that reported for other environmental media where ARGs are typically widespread, including freshwater, soil, wastewater, human gut, and the pig gut (Figure 6). In freshwater, the resistome and ARG‐carrying pathogens (APs) are more abundant in open water than in closed water (i.e., a body of water i.e. not connected to external water bodies), and members of the class Gammaproteobacteria, within the phylum Pseudomonadota, are the dominant ARG hosts [32]. MDRGs were reportedly found in 99.9% of freshwater samples, with their mean relative abundance exceeding 50% across all samples [32]. MDRGs were also the most abundant ARGs in soil, contributing to 70.97% of the total normalized abundance and similar to freshwater, members of the class Gammaproteobacteria were the primary ARG hosts, potentially acting as indicator bacteria of soil contaminants [33]. A total of 345 ARGs were shared between the human and pig gut, with significantly higher ARG abundance in the pig gut than in the human gut. The ratios of ARGs located on plasmids to those located on chromosomes were 1.1 and 0.84 in the pig and human gut, respectively, with tetracycline, vancomycin, and macrolide resistance genes being the most common ARGs in the intestinal environment [34]. Thirteen ARGs were found in wastewater, including specific ARGs such as *mph*(E), *msr*(E), *tet*(A), and *tet*(C), where their spatial and temporal distributions remained consistent globally. For instance, *bla*_*BEL‐2*_ was prevalent in Europe and North America wastewater samples. Additionally, 2102 ARC were recognized as plasmids [35]. In landfill systems, members of Pseudomonadota (258 MAGs, 29.72%) were identified as the primary ARG hosts through whole‐genome recovery. Furthermore, 9419 plasmid‐associated contigs in landfill systems were found to carry ARGs, with a density of 126.4 ARGs per 100 kb (Figure 6).

**Figure 6:**
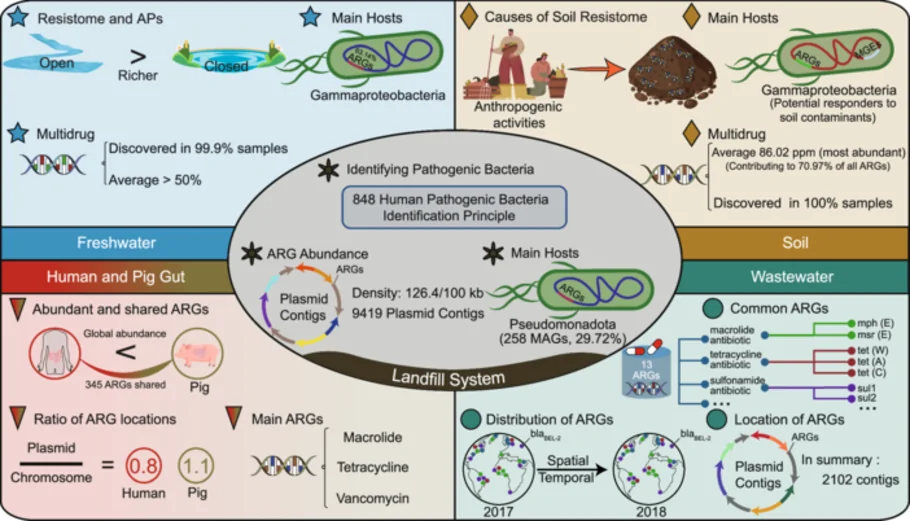
ARG characteristics in various environments on the global scale. In freshwater, the resistome and APs in open water are more abundant than in closed water, with members of the class Gammaproteobacteria serving as primary ARG hosts. Anthropogenic activities are the driving force behind the resistome in soil, with members of the class Gammaproteobacteria serving as the main ARG hosts and acting as potential responders to soil contaminants. ARGs are significantly more abundant in the pig gut than in the human gut, and the plasmid‐to‐chromosome location ratio of ARGs is higher in the pig gut than in the human gut. In wastewater, 13 common ARGs were identified, and their global distribution exhibited no significant temporal variation. AP, ARG‐carrying pathogens; ARG, antibiotic resistance gene; MAG, metagenome‐assembled genome.

## 3 DISCUSSION

The identification, prevention, and control of ARGs in landfills were recently identified in the “Bracing for Superbugs” report by UNEP [5]. Huang et al. emphasized that 96% of ARGs in groundwater may be associated with landfill leachate [36], and studies by Wu et al. and Liu et al. reported that the ARG abundance in landfill leachate decreased with increasing landfill age [37, 38]. Landfill leachate has always been a key focus in assessing the risks of antimicrobial resistance [39]. However, studies have consistently struggled to integrate the various environmental aspects associated with landfills into a comprehensive, holistic evaluation of ARG‐associated risks. In this study, landfill systems served as the primary focus, representing high‐risk environments where numerous interactions occur among living organisms, including humans [19, 24]. This study provides insight into the global landfill resistome, revealing that the ARG diversity, particularly the prevalence of highly mobile MDRGs, is comparable to that observed in heavily polluted environments such as acid mine wastewater and untreated activated sludge [11]. At the MAG level, the primary ARG hosts were identified as members of the phylum Pseudomonadota, including the highly pathogenic strain *P. aeruginosa*. Furthermore, this study established resistome flow pathways into, within, and from landfills. The findings of the study highlight the propagation, evolution, and dissemination of landfill resistomes, emphasizing their importance to public health and safety.

Landfill systems are an underestimated ARG reservoir. Landfill refuse, with an ARG abundance of 1612.5×/Gb, emerged as a considerable hotspot, comparable to other highly contaminated habitats such as acid mine wastewater and untreated sludge, with abundances of 1905.94 and 2013.38×/Gb, respectively (Table S5) [11]. Remarkably, landfill systems harbored 428 ARG subtypes, which was twice as high as the number observed in acid mine wastewater (Table S5) [11]. While the ARG distribution patterns in global wastewater environments are generally considered to be temporally stable [35], this study revealed a significant spatial correlation between ARGs distribution and geographic proximity (*R* = −0.165, *p* < 0.001; Figure 1G and Figure S1B) [40]. Geographical distance imposes limitations on ARG distribution, potentially mediated by differences in waste composition and local antibiotic usage patterns [5]. Andersson et al. demonstrated the selective effect of antibiotic pressure on antibiotic resistance as early as 2010 [41]. Meanwhile, seasonal variations, heavy metal concentrations, and leachate quality parameters are equally important factors influencing ARG distribution [37]. The underlying mechanisms driving this geographic pattern warrant further investigation. Notably, the cross‐environment comparisons using coverage‐based normalization in this study should be interpreted with caution, as they reflect ecological patterns rather than absolute quantitative differences. While this approach revealed consistent ecological patterns across environments, future studies employing single‐copy gene normalization would allow for more stable and comparable relative abundance estimates across samples. For accurate absolute quantification, techniques such as qPCR or digital PCR remain essential and should be integrated with metagenomic sequencing.

MDRGs, the most common ARGs found in landfill systems (32.63% ± 13.60%; Figure 1D,F), were mostly linked to multidrug efflux pumps (73%; Figure 1D,E). Notably, the proportion of MDRGs found in landfill systems was far higher than the 8.8%–25.7% detected in leachate by Jia et al. [42]. This discrepancy may be influenced by specific environmental conditions, as Jia et al. reported that MDRGs dominated treated leachate (64.1%–83.0%) and groundwater (28.7%–76.6%) [42]. In clinical settings, MDRGs are linked to 80% of severe bacterial infections [43]. Although MDRGs were detected in all landfill system samples, their relative abundance was lower than that in other environments, such as freshwater (>50%) and soil (average of 70.97%) (Figure 6) [44, 45].

Within landfill systems, the phylum Pseudomonadota comprised 29.72% of MAGs, with members harboring 47.74% and 56.47% of the total ARGs and MDRGs, respectively. Notably, our previous research demonstrated that the key functional roles of members of the phylum Pseudomonadota in landfills [13, 19, 24, 25], include the conversion of hexavalent chromium, decomposition of cellulose, catalytic oxidation of methane, and remediation of mercury contamination [46, 47, 48].

Resistome movement is highly influenced by horizontal gene transfer (HGT) mediated by MGEs or the genomic location of ARGs (*M* ^2^ = 0.929, *p* < 0.001; Figure 2A,D and Figure S6) [34, 49]. The significant correlation between ARG/MDRG abundance and the presence of MGEs in landfill systems reveals the risk of resistome transmission (*p* < 0.001; Figures S6 and Figure 3B,C). Moreover, the potential risks of ARG‐carrying plasmids represent a critical component within the One Health framework. According to Cao et al., the ratios of ARGs located on plasmid‐associated contigs to those located on chromosome‐associated contigs were 1.1 in the pig gut and 0.8 in the human gut (Figure 6) [34]. By contrast, landfill systems exhibited a much lower plasmid‐to‐chromosome location ratio of 0.34, with 57.3% of ARGs located on chromosomes rather than on plasmids (Figure 2A,B). However, the ARG density on plasmids (126.4 per 100 kb) was approximately 3.5 times higher than that on chromosomes (35.7 per 100 kb) (Figure 2D). This metric reflects ARG‐carrying potential rather than environmental abundance. As for plasmid sequences shorter than 100 kb, the normalization approach provided conservative estimates of ARG‐carrying efficiency. Future studies incorporating copies per cell would provide a more comprehensive assessment of ARG transmission risk in environmental contexts. Nevertheless, given that density (gene‐carrying capacity) and copy number (amplification factor) collectively determine the ecological impact of plasmid‐mediated ARG dissemination, the findings of the study are concerning. More robust evidence, such as quantifying the colocalization of ARGs with MGEs, long‐read sequencing, or using Hi‐C data, is required to validate the results. Furthermore, the number of ARG‐carrying plasmid‐associated contigs in landfill systems (9419) was approximately five times greater than that in global wastewater environments (2102) [35]. Although directly comparing contig numbers has certain limitations owing to differences in research methodologies, ARG density is a critical reference metric for assessing potential risks of HGT. These findings advance the understanding of resistome flow or transmission under the One Health framework.

MDRGs are transmitted via water bodies, agricultural practices, and human activities. Soil, which contains high levels of ARGs, MDRGs, and pathogens [50], exhibited the highest interactions with landfill systems [33], accounting for 34.6% of the total resistome inflow into landfills (Figure 3). The resistome of the soil, as a neighboring environment, frequently enters landfills through phase interactions, while the evolved landfill resistome simultaneously transfers back into the surrounding soil [5]. The pig and human gut (26.6% in total) were also major sources of MDRGs in landfill systems (Figure 3), which is likely due to the disposal of pig and human feces in landfills [51, 52]. Additionally, wastewater introduces MDRGs into landfills, particularly through the disposal of activated sludge [53], while freshwater‐derived MDRGs infiltrate landfills via surface runoff and rainwater penetration [19, 32]. The unknown sources of MDRGs in landfill systems (29.6%) may have included animal intestines from chicken and cattle, as studies by Feng et al. and Liu et al. reported that the intestinal environments of chickens and cattle serve as ARG reservoirs [54, 55]. Meanwhile, waste containing drugs and bacteria can enter landfill systems via MSW streams. Therefore, expanding the source taxonomy to include a broader range of agriculturally important animals is a critical objective for future research as more comprehensive meta‐genomic datasets become available.

Landfills are complex ecosystems with a diverse microbiota, providing optimal conditions for the spread of MDRGs [13, 19, 24, 25], particularly within their solid–liquid–gas phases. The solid phase, primarily composed of landfill refuse, serves as a central reservoir, acting as a source of MDRGs that disseminate into the gas phase (airborne particles) and the liquid phase (leachate) [19, 24]. The high mobility of the gas and liquid phases facilitates substantial interactions and inter‐transfer, with nearly 60% of MDRGs in landfill airborne particles and leachate shared between these phases. Furthermore, correlations between MEGs and MDRGs were stronger within the three‐phase system (*R* ^2^ = 0.171, *p* < 0.001; Figure 3B,C) than across overall landfill systems (*R* ^2^ = 0.096, *p* < 0.001; Figure 3B,C), indicating heightened resistome mobility between these phases. A recent study indicated that 96% of groundwater‐associated ARGs were linked to leachate, directly supporting the high mobility of MDRGs therein [36]. However, this finding requires laboratory‐scale validation, and ARG markers could be employed to track resistome dynamics. In addition to resistome flow pathways into and within landfills, gas emissions and leachate enable resistome dissemination from landfills into the surrounding environment through microbial aerosols, groundwater infiltration, and soil contamination [5].

Herein, two *Pseudomonas* strains were isolated with distinct distribution patterns of genomic islands and MEGs, which significantly differed from those of 33 other *Pseudomonas* strains. This variation may be attributed to the high frequency of HGT in landfills, as evidenced by strain P1_w possessing five times more integration or excision‐related genes than strain P2_w (Figure 5). Furthermore, using the HPB identification principle alongside VFDB annotations revealed landfill refuse as a hotspot for potential pathogenic bacteria harboring multiple AGRs (Figures 1, 4, and Figure S9). Among these, *P. aeruginosa*, a high‐priority pathogen listed by the WHO, was characterized by large numbers of ARGs and genomic islands (Figure S11). *P. aeruginosa* was linked to approximately 560,000 fatalities worldwide in 2019 [27]. In landfills, *P. aeruginosa* can disseminate into the environment through various pathways, including landfill airborne particles, leachate, and excavation [24].

Landfills are essential urban infrastructures that play vital roles in MSW treatment, environmental protection, land reclamation, and public health. However, the findings of the study raise substantial concerns regarding the evolution, movement, and release of extrinsic and intrinsic resistomes from landfill systems into the surrounding environment. This issue is particularly acute in developing countries, where many landfills lack proper sanitary measures, including leachate collection and treatment systems. The study reveals that landfill systems act as sources of diverse and highly mobile ARGs, dominated by MDRGs and pathogenic *Pseudomonas* hosts. These observations emphasize the urgent need for further research into resistome transmission pathways across various environmental media and living organisms, including humans, within and beyond landfill systems. Landfills function as anthropogenic food sources that attract scavengers, with birds, small mammals, and insects serving as active carriers that facilitate resistome dissemination across landfill systems [56]. Open and temporary landfills are highly prevalent in many low‐income countries (LICs) compared to lower‐middle‐income countries (LMICs) (93% vs. 66%) [57], and often located near population centers, greatly increasing the likelihood of ARG‐human interactions [5]. Even in well‐managed landfill systems, landfill leachate harbors substantial populations of antimicrobial‐resistant microorganisms [58]. Despite the adoption of the Global Action Plan on antimicrobial resistance in 2015, few action plans in LICs and LMICs are informed by empirical data or supported with sufficient implementation resources [5, 59]. A genuine One Health approach requires equitable inputs and governance across human, animal, and environmental sectors to effectively address ARG‐associated health threats in policy frameworks. Future governance, planning, and regulatory frameworks should integrate antimicrobial resistance into national development plans, including the environmental sector in One Health coordination to tackle AMR, and ensure that AMR is considered in national climate change planning [5].

## 4 METHODS

### 4.1 Data collection and organization

Data for 161 metagenomic samples associated with landfill systems up to December 3, 2022, were collected from the NCBI database and our lab, representing 21 cities across China, Italy, Canada, Great Britain, Jamaica, and India. This dataset was categorized based on the landfill components spanning the MSW lifecycle, including landfill refuse (*n* = 51), airborne particles (*n* = 6), and leachate (*n* = 38), MSW decomposition and leachate recirculation bioreactors (*n* = 60), and activated sludge from leachate treatment facilities (*n* = 6).

### 4.2 Data analysis and annotation of ARGs

The raw data were subjected to quality control processes, including disassembly, mass shearing, and removal of host‐derived contamination. Following optimization, the processed data were assembled using MEGAHIT bioinformatics software (https://github.com/voutcn/megahit) to generate contigs sequences [60, 61]. ORFs were predicted from contig of ≥500 base pairs (bp) in length using Prodigal software version 2.6.3 (https://github.com/hyattpd/Prodigal). ORFs of ≥100 bp in length were screened and translated into amino acid sequences.

ARG annotation was performed using the DeepARG‐LS model in DeepARG software version 1.0.2 (https://bitbucket.org/gusphdproj/deeparg-ss/src/master/) with default parameters. Contigs containing ARG‐like ORFs were designated as antibiotic resistance contigs (ARCs). The abundances of ARGs and ARCs were calculated using coverage (×/Gb) as the abundance metric. Clean reads from each sample were aligned to the nucleic acid sequences of ARG‐like ORFs using the bwa‐mem algorithm in BWA software version 0.7.17‐r1188 (https://bio-bwa.sourceforge.net/). The alignment process identified the number of ARG‐like ORFs in each sample, from which the abundance (coverage, ×/Gb) of ARG types or subtypes was calculated using Equation 1 [11, 62].

(1) $$\mathrm{Abundance}\left(\mathrm{coverage},\times \mathrm{Gb}\right)\;=\sum_{1}^{n}\frac{N_{\mathrm{mapped reads}}\times \frac{L_{\mathrm{reads}}}{L_{\mathrm{ARG}-\mathrm{like ORF}\;\left(\mathrm{ARC}\right)}}}{S}$$

where *N*_mapped reads_ denotes the number of clean reads matched by ARG‐like ORFs or ARCs; *L*_reads_ refers to the Illumina sequencing read length (150 bp); *L*_ARG‐like ORF (ARC)_ represents the sequence length of target ARG‐like ORFs or ARCs (in bp); *n* denotes the number of ARG‐like ORFs or ARCs belonging to the same classification; and *S* refers to the size of the corresponding metagenomic clean dataset (in Gb). Notably, normalized ARG abundance is typically expressed as copies per cell in environmental studies [63]. However, coverage (×/Gb) was comprehensively selected as the abundance metric for ARGs in this study based on data accessibility, methodological consistency, and comparative integrity. This approach will hopefully provide insight for future research assessing ARG risks within the One Health framework.

### 4.3 ARG mobility characteristics and annotation

The number of ARCs on plasmids or chromosomes was predicted using PlasFlow software version 1.1 (https://github.com/smaegol/PlasFlow) Differences in the potential mobility of ARGs across different samples were determined by analyzing the predicted genetic locations of ARCs [64]. Notably, the minimum contig length in PlasFlow is 1000 bp, and PlasFlow exhibits some identification bias toward short contigs. However, PlasFlow reportedly achieves 96% accuracy in identifying plasmids and chromosomes [64]. Mobile genetic elements (MGEs) in contigs were compared using MGEs90 software (https://bench.cs.vt.edu/ftp/data/databases/MGEs90.fasta), also known as NanoARG‐MGE. MGE‐like ORFs were determined by aligning the amino acid sequences of ARC‐associated ORFs against the MGEs90 database using blastp in DIAMOND software version 2.0.15.153 using the following criteria: e‐value ≤ 1 × 10^−5^, identity ≥80%, and coverage ≥70%. Abundance was calculated for MGE‐like ORFs using coverage (×/Gb) as the abundance metric. To further assess the potential mobility of ARGs, co‐occurrence relationships were identified when ARGs and MGEs were located on the same contig [14, 64, 65].

### 4.4 Traceability analysis of MDRGs in landfill systems

SourceTracker2 [22] was employed to elucidate the potential sources of MDRGs within landfill systems. First, external contributors of MDRGs in landfill systems were identified, wherein landfill systems were treated as “sink” environments, while freshwater, the human and pig gut, soil, and wastewater were defined as potential “sources.” Second, MDRG flow within landfill systems was explored, wherein landfill refuse, leachate, and airborne particles were considered as “source” and “sink” environments to evaluate MDRG exchanges among these landfill components. To eliminate depth‐dependent bias, source and sink count tables were rarefied to the minimum library size via scaling with ranked subsampling with a fixed seed (123) prior to SourceTracker2 analysis. For both steps, SourceTracker2 analysis was replicated five times using default settings, and the source contribution proportions were averaged to obtain reliable estimates.

### 4.5 Genome reconstruction and annotation

MAGs were derived through the process of single‐sample binning, which involved constructing an index to facilitate the alignment of cleaned sequence reads to assembled contigs using Bowtie2 software version 2.3.4.1. Contigs of >1000 bp in length were independently assembled using two binning tools: Semibin2 software version 1.5.1 and MetaBAT2 software version 2.15. MAGs were subsequently refined using DAS tool version 1.1.6 to synthesize the data obtained using both binning methods. The integrity and purity of MAGs were assessed using CheckM2 tool version 1.0.12, and those with >90% completeness and <5% contamination were classified as high‐quality MAGs. Dereplication of high‐quality MAGs was performed using dRep software version 3.4.4 with default settings to eliminate redundancy at the species level, yielding 1537 unique, high‐quality MAGs.

The high‐quality MAGs were taxonomically categorized using GTDB‐Tk toolkit version 2.3.2. The relative abundance of each MAG was determined using CoverM tool version 0.6.1. ARGs within MAGs were identified using DeepARG software.

### 4.6 Isolation of *Pseudomonas* spp. strains

Landfill leachate samples were collected on December 24, 2018 in seven Chinese cities (Chendu, Shenzhen, Changsha, Tangshan, Chongqing, Shanghai, and Guangzhou), as previously described [66], which were pooled to maximize the recovery of *Pseudomonas* diversity, as intercity comparison was not the objective of the isolation step. A 1 mL aliquot of the mixed leachate samples was diluted in 50 mL of sterile phosphate‐buffered saline (pH 7.4), followed by serial dilutions to 10^−1^, 10^−2^, and 10^−3^. To prepare *Pseudomonas* selective agar medium, 16 g gelatin, peptone, 10 g enzymatic casein digest, 10 g potassium sulfate, 1.4 g magnesium chloride, and 11 g agar (pH 7.1) were dissolved in 1 L of water, the mixture was sterilized, cooled to 45°C, and 1 mL of sterilized CFC and 1 mL of sterilized glycerol were added to 1 L of sterilized medium. Subsequently, 200 µL of each dilution was plated onto Pseudomonas selective agar medium. Three *Pseudomonas* spp. strains were isolated from the leachate samples. In addition, 18 landfill waste samples were collected on October 19, 2018 at the Xiaping landfill in Shenzhen, China. Sample selection was based on landfill age (see Table S6 for definitions of sample names). Based on the collection depth of the samples, 8 g each of QY‐2 and CX2‐2‐2 were mixed, while 8 g each of the remaining 16 samples were similarly combined. These waste mixtures underwent the same treatment process as the leachate samples, resulting in the isolation of two additional *Pseudomonas* spp. strains.

DNA was extracted from *Pseudomonas* spp. strains and de novo sequencing was performed. The scanning map was sequenced using the Illumina Novaseq‐X‐plus PE150 platform. For DNA samples that passed quality checks, ~400 bp insert fragments were constructed, followed by double‐end and single‐end sequencing with 150 bp read lengths. These reads were assembled into multiple genome scaffolds. The completion map was sequenced using Illumina and PacBio sequencing platforms. Sequencing and quality control processes were performed by Majorbio Biopharma Technology in Shanghai, China. Based on genome screening criteria (completeness > 90% and contamination < 5%), only L2, L3, and S1 from PRJNA796178 were retained, corresponding to P_1_w, P_2_w, and P_3_w, respectively. The original whole‐genome sequences were uploaded to NCBI under accession number PRJNA796178, including detailed descriptions of the sampling sites.

### 4.7 Genome structure annotation

The phylogenetic tree was visualized using the iTOL platform [67]. Thirty‐five *Pseudomonas* spp. strains were retained after applying screening criteria of > 90% genome completeness and <5% contamination (Table S2). Genomic islands in *Pseudomonas* spp. were predicted using the IslandViewer4 tool, and their locations were visualized using the Proksee tool. Annotation within Proksee was performed using the mobileOG‐db database and CARD Resistance Gene Identifier tool with default parameters. Additionally, VFs were annotated using the VFDB database.

### 4.8 HPB identification principle

A list of HPBs was compiled after reviewing the literature and databases [4, 11, 68, 69, 70], with updates and revisions to the species names (Table S7), including the WHO's 2024 priority pathogen lists, ESKAPE priority pathogen lists, and other common pathogens. This HPB list, comprising 848 species‐level HPBs, was employed to identify potential pathogenic bacterial genomes among MAGs. Representative whole‐genome sequences of these 848 HPBs were gathered from the NCBI RefSeq database to create an HPB sequence database. Potential pathogenic MAGs were identified by comparing their species annotation with the HPB list and identifying the presence of VFs through VFDB annotation. MAGs were designated as potentially pathogenic if their species annotation matched a name on the list and contained VFs. Finally, the whole‐genome sequences of the identified HPBs and those in the original database were uploaded to https://github.com/hongwenqing/HPB-identification-principle.

### 4.9 Graphical visualization and statistical analysis

Graphs were visualized using R version 3.6.3, RStudio version 1.1.463, and online OmicShare tools (https://www.omicshare.com/tools). Beta‐diversity was analyzed based on Bray–Curtis dissimilarity matrices calculated directly from the ARG abundance data (×/Gb). The homogeneity of the data's dispersion was tested using the betadisper method. Community composition differences were visualized using principal coordinate analysis (PCoA). Permutational multivariate analysis of variance (PERMANOVA) with 999 permutations was then performed via the adonis function to test the statistical significance of the grouping factor on the observed variation. The Kruskal‐Wallis test was used for multiple comparisons of ARG abundance across plasmids and of MGE abundance across landfill components, followed by post hoc pairwise comparisons using the Wilcoxon test. Then, Tukey's test was utilized for PC1 comparisons during PCoA analysis. Meanwhile, we divided the 161 samples into test and training sets, then evaluated the importance of each ARG in predicting total abundance by constructing 500 decision trees in a random forest.

## AUTHOR CONTRIBUTIONS

Wenqing Hong performed the conceptualization, methodology, investigation, and wrote the original draft. Rui Zhang contributed to the conceptualization, methodology, and wrote the original draft. Xiaoxing Lin conducted the conceptualization, methodology, and investigation. Yangqing Wang contributed to the conceptualization, methodology, and reviewed the manuscript. Shu Yang was responsible for the conceptualization, methodology, investigation, visualization, funding acquisition, project administration, and reviewed the manuscript. Farees ud din Mufti and Yuping Yao performed the methodology and investigation. Guowei Zhou reviewed and edited the manuscript. Chuanwu Xi supervised the study and reviewed the manuscript. Weixi Shu contributed to the conceptualization, supervised the study, and reviewed the manuscript. Gordon Price and Youcai Zhao supervised the study and reviewed the manuscript. Liyan Song performed the conceptualization, methodology, investigation, funding acquisition, project administration, supervision, and reviewed the manuscript. All authors have read the final manuscript and approved it for publication.

## CONFLICT OF INTEREST STATEMENT

The authors declare no conflicts of interest.

## ETHICS STATEMENT

No animals or humans were involved in this study.

## Supporting information


**Figure S1.** Global distribution pattern of ARGs.
**Figure S2.** ARG subtype principal coordinate analysis (PCoA) analysis, multigroup difference test by Tukey.
**Figure S3.** The top 12 ARG subtypes by abundance (coverage, ×/Gb), with other ARG subtypes grouped under “Others”.
**Figure S4.** The screening of potential indicator ARGs in landfill systems using a random forest model.
**Figure S5.** The abundance of the potential indicator gene *mexA* explains 55.2% of the variation in total ARG abundance across landfill systems (*p* < 0.001).
**Figure S6.** Procrustes analysis reveals a significant correlation between ARG subtype composition and MGE subtype composition (M² = 0.929, *p* = 0.001).
**Figure S7.** Comparison of average connection strength between source environments and landfill.
**Figure S8.** Distribution patterns of (A) total ARGs and (B) the potential indicator gene *mexA* across the three phases of the landfill environment.
**Figure S9.** Abundance of human pathogenic bacterial MAGs in landfill systems, as determined by the Human Pathogenic Bacteria (HPB) Identification Principle.
**Figure S10.** Abundance of mobile genetic elements (MGEs) within MAG genomes, summarized according to the phylum of the corresponding MAG.
**Figure S11.** Categorical gene counts across 35 *Pseudomonas* spp. genomes (including human gut and landfill system), with variance assessed by the Wilcoxon test.
**Figure S12.** COG (Clusters of Orthologous Genes) annotation results of the *Pseudomonas aeruginosa* genome from landfill systems.
**Figure S13.** Gene count distribution in the *Pseudomonas aeruginosa* genome, with variance assessed by the Wilcoxon test.

**Table S1.** Information on 161 metagenomic samples from global landfill systems.
**Table S2.** Quality information for all filtered MAGs (contamination level, completeness, and species annotation).
**Table S3.** Information on Metagenome‐Assembled Genomes of 35 *Pseudomonas* spp. strains.
**Table S4.** Antibiotic resistance genes specific to 35 strains of *Pseudomonas* spp. in landfill systems and the human gut.
**Table S5.** Abundance and number of ARGs in freshwater sediments, acid mine wastewater and untreated sludge.
**Table S6.** Name of landfill refuse sample (Xiaping Landfill, Shenzhen, China).
**Table S7.** 848 HPB identification principle.

## Data Availability

The data underlying this article are available in the article and in its online supplementary material. Specifically, the metagenomic data used in this study is the “landfill system dataset.” It consists of 161 metagenomic samples from landfills around the world, including 43 samples from our laboratory and 118 samples from public databases (Table S1). The raw data can all be found in NCBI (https://www.ncbi.nlm.nih.gov/bioproject/PRJNA590620/, https://www.ncbi.nlm.nih.gov/bioproject/?term=PRJNA788122, https://www.ncbi.nlm.nih.gov/bioproject/?term=PRJNA412592, https://www.ncbi.nlm.nih.gov/bioproject/?term=PRJNA438425), and the project numbers for each data are listed in Table S1. Meanwhile, the “848 HPB Identification Principle Database” we built has been uploaded to GitHub (https://github.com/hongwenqing/HPB-identification-principle). Lastly, the original whole‐genome sequences have been uploaded to NCBI (https://www.ncbi.nlm.nih.gov/bioproject/?term=PRJNA796178%20). The data and scripts used are saved in GitHub https://github.com/hongwenqing/Resistome-flow-in-global-landfill-systems. Supplementary materials (figures, tables, graphical abstract, slides, videos, Chinese translated version, and update materials) may be found in the online DOI or iMeta Science http://www.imeta.science/imetaomics/.
