# Supplementary material for: Resistome flow in global landfill systems
Source: IMetaOmics. 2026 Jan 6;3(1):e70075. doi: 10.1002/imo2.70075 (PMC13674416; doi:10.1002/imo2.70075)
Supplement: Supplementary file 1 [file IMO2-3-e70075-s001.docx]

**Supporting information to**

**Resistome flow in global landfill systems**

**Running title**: Global landfill system resistome profile

Wenqing Hong^1#^, Rui Zhang^1#^, Xiaoxing Lin^2^, Yangqing Wang^3^, Shu Yang^4^, Farees ud din Mufti^1^, Yuping Yao^1^, Guowei Zhou^1^, Chuanwu Xi^5^, Weixi Shu^6^, Gordon Price^6^, Youcai Zhao^7^, Liyan Song^1,3,6^*

^1^School of Resources and Environmental Engineering, Anhui University, Hefei 230601, China

^2^School of Engineering, Westlake University, Hangzhou 310030, China

^3^Chongqing Institute of Green and Intelligent Technology, Chinese Academy of Science, Chongqing 400714, China

^4^Institute of Public Health Sciences, Division of Life Science and Medicine, University of Science and Technology of China, Hefei 230026, China

^5^Department of Environmental Health Sciences, University of Michigan, Michigan 48109, USA

^6^Department of Engineering, Dalhousie University, Nova Scotia NS, B2N 5E3, Canada

^7^The State Key Laboratory of Pollution Control and Resource Reuse, School of Environmental Science and Engineering, Tongji University, Shanghai 200092, China

^#^These authors contributed equally: Wenqing Hong, Rui Zhang

*Correspondence: [20023@ahu.edu.cn](mailto:20023@ahu.edu.cn) (Liyan Song)

**Figure S1 Global distribution pattern of ARGs.** (A) Distribution of 161 metagenomic samples. 161 metagenomic samples were collected from the NCBI database and our lab, representing 21 cities across China, Italy, Canada, Great Britain, Jamaica, and India. (B) Distribution pattern of Chao and Shannon indices of ARGs with longitude.

**Figure S2 ARG subtype principal coordinate analysis (PCoA) analysis, multigroup difference test by Tukey.** The distribution of ARG subtypes across different groups showed significant differences based on Adonis test (*p* < 0.05). The distribution of PC1 values varied considerably among groups, with Group A and AS Group exhibiting relatively concentrated distributions.

**Figure S3** **The top 12 ARG subtypes by abundance (coverage, ×/Gb), with other ARG subtypes grouped under “Others”.** Among these, the most abundant were rpoB2 (7.71 ± 4.83%), ompR (4.88 ± 2.61%), and ArlR (4.50 ± 1.94%). R, landfill refuse; A, landfill airborne particles; B, MSW decomposition and leachate recirculation bioreactors; L, landfill leachate; AS, activated sludge from leachate treatment facilities.

**Figure S4 The screening of potential indicator ARGs in landfill systems using a random forest model.** The results indicate that *mexA* contributed most significantly to the variation in ARG abundance, with the model achieving an R² of 0.893, the root mean square error (RMSE) of 283.1, and the mean absolute error (MAE) of 195.1.

**Figure S5 The abundance of the potential indicator gene *mexA* explains 55.2% of the variation in total ARG abundance across landfill systems (*p* < 0.001).**

**Figure S6 Procrustes analysis reveals a significant correlation between ARG subtype composition and MGE subtype composition (M² = 0.929, *p* = 0.001).**

**Figure S7 Comparison of average connection strength between source environments and landfill.** The bar plot displays the mean connection strength calculated for each source environment-landfill pair based on shared MDRG subtypes. Connection strength was computed as the geometric mean of MDRG abundances ($\sqrt{\left( source abundance\times landfill abundance \right)}$) for each shared antimicrobial resistance gene, then averaged across all common MDRGs between each source and the landfill sink. The values represent the average strength of resistome connections, with soil demonstrating the strongest association (121.5), followed by pig gut (116.6), human gut (75.0), wastewater (60.5), and freshwater (24.9).

 **Figure S8 Distribution patterns of (A) total ARGs and (B) the potential indicator gene *mexA* across the three phases of the landfill environment.** R, landfill refuse; A, landfill airborne particles; L, landfill leachate.

**Figure S9 Abundance of human pathogenic bacterial MAGs in landfill systems, as determined by the Human Pathogenic Bacteria (HPB) Identification Principle.** R, landfill refuse; A, landfill airborne particles; B, MSW decomposition and leachate recirculation bioreactors; L, landfill leachate; AS, activated sludge from leachate treatment facilities.

**Figure S10 Abundance of mobile genetic elements (MGEs) within MAG genomes, summarized according to the phylum of the corresponding MAG.**

**Figure S11 Categorical gene counts across 35 *Pseudomonas* spp. genomes (including human gut and landfill system), with variance assessed by the Wilcoxon test.** The *p*-value for the between-group difference test is indicated by a horizontal line between the two sets of data. A *p*-value < 0.05 indicates a statistically significant difference between the two groups.

**Figure S12 COG (Clusters of Orthologous Genes) annotation results of the *Pseudomonas aeruginosa* genome from landfill systems.**

**Figure S13 Gene count distribution in the *Pseudomonas aeruginosa* genome, with variance assessed by the Wilcoxon test.** The *p*-value for the between-group difference test is indicated by a horizontal line between the two sets of data. A *p*-value < 0.05 indicates a statistically significant difference between the two groups.
